# Adaptive Strategies of Desert Shrub Stem–Leaf Anatomical Traits in the High-Altitude Qaidam Basin

**DOI:** 10.3390/plants15081213

**Published:** 2026-04-15

**Authors:** Yuanyuan Wang, Siyu Liu, Chengjun Ji

**Affiliations:** 1 Key Laboratory of Vegetation and Environmental Change, Institute of Botany, Chinese Academy of Sciences, Beijing 100093, China; wangyuanyuan@ibcas.ac.cn; 2 Hebei Normal University Library, Hebei Normal University, Shijiazhuang 050024, China; liusylib@hebtu.edu.cn; 3 Institute of Ecology, College of Urban and Environmental Sciences, and State Key Laboratory for Vegetation Structure, Function and Construction (VegLab), Peking University, Beijing 100871, China

**Keywords:** trait coordination, hydraulic safety, high-altitude dry ecosystems, multiple stresses

## Abstract

High-altitude arid regions are characterized by concurrent water scarcity, low temperatures, and intense solar radiation. However, the adaptive mechanisms of desert shrubs to these combined stressors remain poorly understood. To address this gap, we integrated large-scale field surveys with laboratory measurements of eight stem and leaf anatomical traits across six common desert shrub species in the Qaidam Basin. Principal component analysis (PCA) revealed two primary axes of trait variation. The first principal component (PC1) characterized a trade-off between leaf protective traits (e.g., cuticle and epidermal thickness) and stem hydraulic-storage traits (e.g., central cylinder, xylem, and pith diameters). The second principal component (PC2) was primarily loaded by stem cortex thickness, representing a physiological buffering mechanism. Based on PC1, species were categorized into two distinct strategic groups. Group A prioritized investment in stem conductive and storage tissues, enhancing hydraulic safety under hotter, high-evaporative demand conditions. Conversely, Group B exhibited reinforced leaf protective structures, consistent with tolerance to high radiation and low-temperature stress at higher elevations. The environmental gradients were the primary drivers of this divergence: Group A was associated with aridity, whereas Group B was correlated with elevation. Our findings demonstrate that desert shrubs in the Qaidam Basin have employed diverse adaptive strategies via the modulation of organ-specific anatomical traits to mitigate environmental stressors. These findings offer valuable insights into plant adaptive mechanisms, with implications for predicting vegetation responses and informing ecological restoration in high-altitude arid ecosystems.

## 1. Introduction

Arid ecosystems, which cover approximately 41% of the global land surface [1,2], represent a critical component of the terrestrial biosphere. These ecosystems are increasingly threatened by the combined pressure of climate warming, altered precipitation regimes, land-use intensification, and overexploitation [3]. Under the persistent influence of these drivers, arid regions are projected to expand to nearly 50% of Earth’s land surface by the end of this century [4,5], potentially amplifying risks to biodiversity, carbon storage, and ecosystem stability. Desert shrubs play a vital role in sustaining the structure and function of arid ecosystems by stabilizing sand, reducing erosion, conserving soil water content, sequestering carbon, and supporting biodiversity [1,4,6]. As the resistance and recovery of arid ecosystems depend heavily on the persistence of these species, uncovering the mechanisms by which desert shrubs adapt to environmental stress is critical. Such knowledge is needed not only to understand plant survival in extreme environments but also to improve predictions of vegetation dynamics and to guide restoration and sustainable management in a rapidly expanding dryland area.

Plant functional traits provide a powerful framework for linking individual plant adaptation strategies to ecosystem functions [7]. They capture how species acquire, allocate, and conserve resources, and they often converge along coordinated spectra reflecting trade-offs between growth and stress tolerance [8,9,10]. In dryland ecosystems, however, trait-based studies have predominantly emphasized morphological or chemical traits, such as specific leaf area, leaf dry matter content, or leaf nitrogen concentration [11,12,13,14], while paying far less attention to the anatomical structures that underpin these trait expressions. This bias limits mechanistic inference, because anatomy directly determines hydraulic transport, tissue protection, mechanical support, and carbon assimilation, functions that are central to survival in desert environments [15,16,17]. For desert shrubs in particular, whose persistence depends on coping with multiple concurrent stresses, anatomical traits may provide the clearest window into how resource investment is coordinated across organs. A trait-based understanding of desert plant strategies is therefore needed to explicitly integrate anatomical structures to explain the mechanisms of stress tolerance in arid ecosystems.

Existing research on the stem and leaf anatomical traits of desert shrubs has largely examined responses to single stressors, such as drought or salinity stress, and thus offers only a preliminary understanding of plant adaptation in arid environments [18,19]. Reports of shifts in traits related to gas exchange, water transport, and resource acquisition, such as stomatal density and size, as well as root length and diameter, suggest that desert shrubs can adjust structural features to reduce water loss or maintain hydraulic safety [20,21]. However, in high-altitude desert regions, plants face multiple concurrent stresses, including water limitation, low temperatures, and intense solar radiation [22]. These combined stresses may impose coordinated selection on anatomical traits, potentially generating integrated adaptive strategies rather than isolated trait responses. Whether such multi-stressor environments drive anatomical differentiation among coexisting shrubs, and whether these differences can be resolved into distinct functional groups, remains poorly understood.

High-altitude deserts offer a natural laboratory for testing how multiple environmental stresses shape plant anatomical strategies. The Qaidam Basin, located on the northern Tibetan Plateau at elevations exceeding 3000 m, ranks among the world’s highest inland basins [23]. Extreme aridity, intense radiation, and large diurnal temperature variation combine to impose both water limitation and cold stress, generating steep climatic and edaphic gradients across relatively short spatial distances. This setting offers a powerful opportunity to examine how anatomical traits are coordinated with environmental variation. Here, we aimed to identify (i) whether co-occurring desert shrubs exhibit distinct anatomical traits corresponding to alternative adaptive strategies in high-altitude arid environments, and (ii) whether the primary environmental drivers differ among these strategies. To achieve these goals, we combined large-scale transect surveys with detailed laboratory measurements of stem and leaf anatomical traits of six dominant shrub species. Integrating these anatomical data with climatic and soil variables allowed us to test how structural investment patterns mediate shrub adaptation under multi-stressor conditions. By linking anatomical differentiation to environmental filtering, our results provide new insight into how high-altitude desert plants organize stress tolerance strategies and how environmental gradients shape functional group distribution.

## 2. Results

### 2.1. Stem and Leaf Anatomical Traits of Desert Shrubs

**Figure 1:**
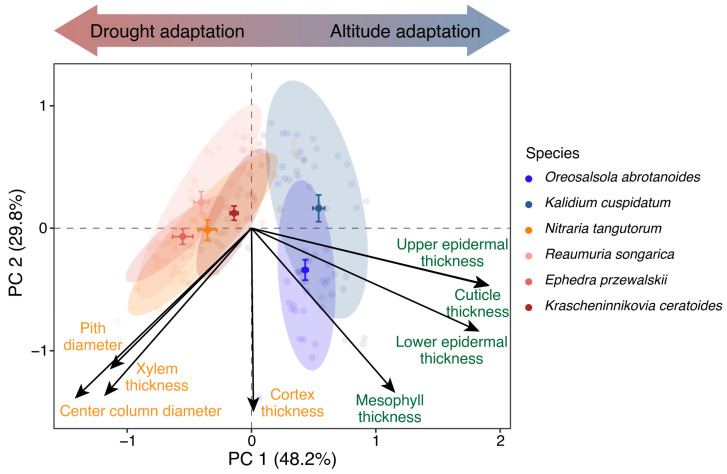
Principal component analysis (PCA) for stem and leaf anatomical traits in desert shrubs.

To characterize covariation in stem and leaf anatomical traits across six desert shrub species, we first conducted a principal component analysis (PCA) based on eight measured anatomical traits (Figure 1). These comprised four stem traits—cortex thickness (CT), central cylinder diameter (CCd), xylem thickness (XT), and pith diameter (PD), and four leaf traits—cuticle thickness (Ct), upper epidermal thickness (ET_up_), lower epidermal thickness (ET_low_), and mesophyll thickness (or assimilation branch parenchyma thickness) (MT). All variables were standardized prior to PCA.

The first two principal components extracted by PCA collectively accounted for 78% of the total variance, capturing the dominant patterns of anatomical differentiation among species. The first principal component (PC1) explained 48.2% of the total variance, with strong positive loadings dominated by leaf anatomical traits (MT, ET_up_, ET_low_, Ct) and strong negative loadings primarily associated with stem anatomical traits (CCd, PD, XT). The second principal component (PC2) accounted for 29.8% of the total variance, with stem CT as the key negatively loaded trait. Species were clearly separated along PC1. *Nitraria tangutorum*, *Reaumuria songarica*, *Ephedra przewalskii* and *Krascheninnikovia ceratoides* were mainly distributed on the negative side of PC1, whereas *Kalidium cuspidatum* and *Oreosalsola abrotanoides* were primarily located on the positive side of PC1. In contrast, species showed broad overlap along PC2, with no clear interspecific separation. Based on their positions along PC1, we therefore classified the four species located on the negative side of PC1 as Group A (*N. tangutorum*, *R. songarica*, *E. przewalskii*, and *K. ceratoides*), and the two species located on the positive side of PC1 as Group B (*K. cuspidatum* and *O. abrotanoides*).

### 2.2. Variation in Stem and Leaf Anatomical Traits Among Species

**Figure 2:**
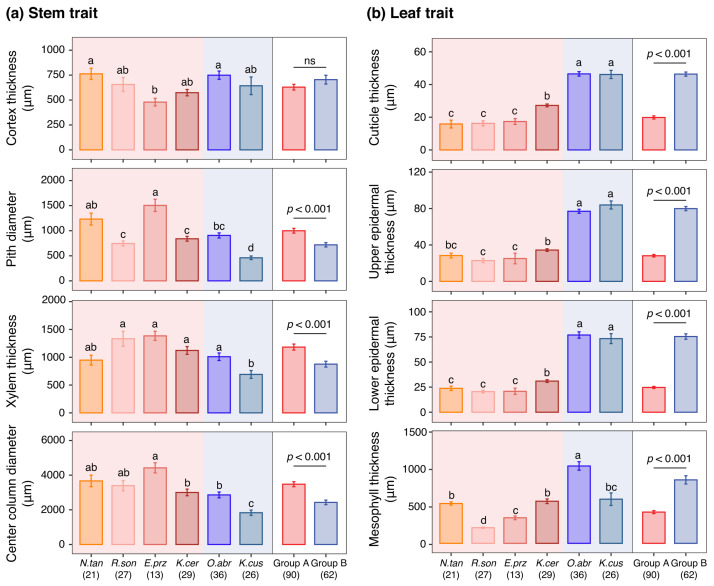
Variation in stem (**a**) and leaf (**b**) anatomical traits among six desert shrub species and between two functional groups. Different lowercase letters above bars indicate significant differences among species for each trait (*p* < 0.05). "ns" denotes not significant. Numbers in parentheses denote the number of sampling replicates across the transect for each species or functional group. Red and blue shadings denote Groups A and B, respectively. Species abbreviations: *N.tan*, *Nitraria tangutorum*; *R.son*, *Reaumuria songarica*; *E.prz*, *Ephedra przewalskii*; *K.cer*, *Krascheninnikovia ceratoides*; *O.abr*, *Oreosalsola abrotanoides*; *K.cus*, *Kalidium cuspidatum*.

One-way analysis of variance (ANOVA) revealed significant differences in both stem and leaf anatomical traits among the six species (Figure 2). For stem traits, the PD, XT, and CCd of *E. przewalskii* were greater than those of the other species, whereas its CT was lower (*p* < 0.001). In contrast, *K. cuspidatum* showed significantly lower PD, XT, and CCd values compared to the remaining species (Figure 2a). For leaf traits, *K. cuspidatum* and *O. abrotanoides* exhibited significantly higher values for Ct, ET_up_, ET_low_ and MT compared with the remaining species (Figure 2b).

Intergroup comparisons demonstrated that Group A showed significantly greater values for three stem-related traits (PD, XT, and CCd) compared with Group B (Figure 2a). In contrast, Group B had significantly higher values for four leaf-related traits (i.e., Ct, ET_up_, ET_low_, and MT) than Group A (Figure 2b). No significant difference in CT was observed between the two functional groups (*p* > 0.05).

### 2.3. Relationships Between Dominant Traits of Desert Shrubs and Environmental Factors

**Figure 3:**
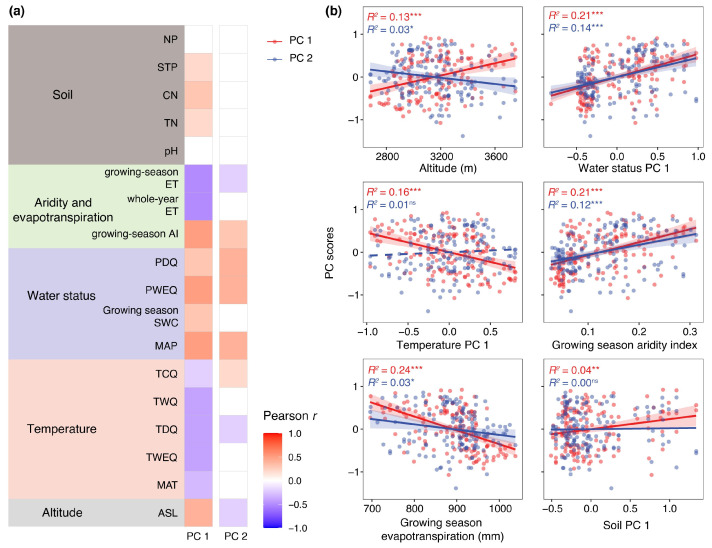
Contributions of environmental factors to anatomical variation. (**a**) Pearson correlations between trait principal components (PC1 and PC2) and key climatic, edaphic, and water-related variables. (**b**) Relationships between trait PCs and integrated environmental gradients represented by the first principal component of temperature-related variables (Temperature PC1), moisture-related variables (Moisture PC1), and soil variables (Soil PC1). Significance levels: * *p* < 0.05, ** *p* < 0.01, *** *p* < 0.001; ‘ns’ = not significant. Red circles, lines, and shading denote PC1, and blue ones represent PC2. Abbreviations—Soil properties: NP, nitrogen-to-phosphorus ratio; STP, soil total phosphorus; CN, carbon-to-nitrogen ratio; TN, total nitrogen; pH, soil pH. Aridity and evapotranspiration: Growing-season ET, evapotranspiration; Al, aridity index. Water status: PDQ, precipitation of the driest quarter; PWEQ, precipitation of the wettest quarter; SWC, growing season soil water content; MAP, mean annual precipitation. Temperature: TCQ, mean temperature of the coldest quarter; TWQ, mean temperature of the warmest quarter; TDQ, mean temperature of the driest quarter; TWEQ, mean temperature of the wettest quarter; MAT, mean annual air temperature.

To quantify environmental controls on anatomical differentiation, we correlated species scores along Trait PC1 and PC2 with climatic and soil variables using Pearson’s correlation analysis (Figure 3a). PC1 scores showed significant associations with elevation; multiple temperature-related variables (all *p* < 0.05), including mean annual temperature (MAT), temperature of the warmest quarter (TWEQ), temperature of the driest quarter (TDQ), temperature of the warmest quarter (TWQ), and temperature of the coldest quarter (TCQ); water-availability metrics, including mean annual precipitation (MAP), growing-season soil water content (SWC), precipitation of the wettest quarter (PWEQ), and precipitation of the driest quarter (PDQ); as well as aridity index, evapotranspiration (ET), and soil nutrient indicators (total nitrogen, TN; C:N ratio, CN; and total phosphorus, TP). In contrast, PC2 exhibited a more restricted environmental association. It correlated significantly with elevation, selected water-related variables (MAP, PWEQ, PDQ), aridity index, and growing-season ET (all *p* < 0.05), but showed no significant relationship with temperature variables or soil nutrient indicators (Figure 3a).

As climatic and edaphic variables are often collinear, we further conducted separate PCAs for temperature-related variables, moisture-related variables, and soil nutrient variables. The first principal component of each environmental category explained 85.0%, 78.6%, and 46.4% of the total variance, respectively, and was used as an integrated descriptor of temperature, moisture, and soil conditions. Regression analyses using these composite environmental axes yielded patterns consistent with the pairwise correlations (Figure 3b). Trait PC1 was significantly associated with temperature PC1, water status PC1, and soil PC1, indicating coordinated responses of anatomical traits to thermal, hydric, and nutrient gradients (all *p* < 0.05). In contrast, trait PC2 was significantly related to water status PC1 and showed no relationship with temperature or soil PC1 (Figure 3b).

Partial correlation analyses revealed that the association between Trait PC1 and elevation was robust despite environmental mediation (Table 1). Without controlling for covariates, Trait PC1 was strongly correlated with elevation (*p* < 0.001). This relationship remained significant after controlling individually for mean annual precipitation (MAP; *p* < 0.001), aridity index (*p* < 0.001), and soil nutrient variables (TN, TP, and C:N ratio; *p* = 0.004). When multiple water status-related variables were controlled simultaneously (MAP + PWEQ + PDQ + SWC), the correlation between Trait PC1 and elevation weakened but remained significant (*p* = 0.017). Controlling for mean annual temperature (MAT) also reduced the strength of the relationship (*p* = 0.049), and inclusion of composite temperature variables (MAT + TWEQ + TDQ + TCQ) rendered the association marginal (*p* = 0.067). In contrast, controlling for evapotranspiration (ET) eliminated the elevational association entirely (*p* = 0.424).

### 2.4. Environmental Differentiation Between Anatomical Groups

**Figure 4:**
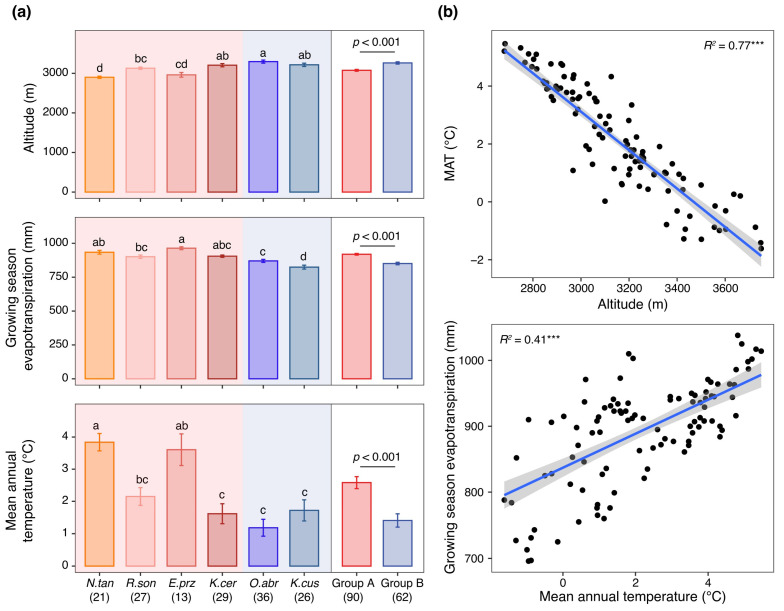
Environmental differentiation between anatomical groups. (**a**) Comparisons of elevation, growing season evapotranspiration (Growing-season ET), and mean annual temperature (MAT) across plots occupied by Group A and Group B species. Different lowercase letters above bars indicate significant differences among species for the elevation, Growing-season ET and MAT (*p* < 0.05). Red and blue shadings denote Groups A and B, respectively. (**b**) Correlations among elevation, Growing-season ET, and MAT across the study region. Circles denote individual sampling plots. Solid lines are linear regression fits. Gray shadings indicate the 95% confidence bands of the regression lines. “***” indicates statistical significance at *p* < 0.001. Numbers in parentheses denote the number of sampling replicates across the transect for each species or functional group.

We compared elevation, growing season ET, and MAT across plots occupied by Group A and Group B species (Figure 4a). Clear environmental differentiation emerged between the two anatomical groups. Plots supporting Group A species occurred at lower elevations than those supporting Group B species. In particular, *N. tangutorum* occupied the lowest elevations (2900.9 ± 27.7 m), positioning Group A toward the lower end of the elevational gradient. Growing-season ET showed the opposite pattern. Group A plots experienced higher Growing-season ET than Group B plots. Within Group B, *O. abrotanoides* and *K. cuspidatum* occurred in environments with substantially lower Growing-season ET (870.3 ± 11.0 mm and 824.1 ± 14.1 mm, respectively) compared with the Group A mean (919.1 ± 6.2 mm). Mean annual temperature further reinforced this separation. Although the numerical difference was modest, Group A plots exhibited higher MAT than Group B plots.

Across the study region, elevation and temperature were negatively correlated, such that temperature declined with increasing altitude (Figure 4b). In contrast, temperature and Growing-season ET were positively correlated, indicating that warmer sites experienced stronger evaporative demand (Figure 4b).

## 3. Discussion

### 3.1. Different Functional Groups and Adaptive Strategies of Desert Shrubs

Principal component analysis of stem and leaf anatomical traits revealed two orthogonal axes of anatomical differentiation among the six desert shrub species (Figure 1). The PC1 axis segregated leaf anatomical traits (MT, ET_up_, ET_low_, Ct) from stem anatomical traits (CCd, PD, XT) at opposite poles. Leaf traits loaded positively, whereas stem hydraulic traits loaded negatively, indicating a coordinated but opposing allocation pattern between leaf protective tissues and stem conductive and storage architecture. Functionally, PC1 therefore captures an axis of resource allocation between leaf-level protection and transpiration control (e.g., defense and transpiration control) versus stem-level water transport and storage. According to species ordination along the PC1 axis, shrubs can be classified into two functional groups. Group A, including *N. tangutorum*, *R. songarica*, *E. przewalskii*, and *K. ceratoides*, distributed along the negative direction of PC1, tended to sustain efficient water transport by enhancing the stem hydraulic architecture and water storage capacity [24]. Group B, comprising *K. cuspidatum* and *O. abrotanoides*, distributed along the positive direction of PC1, adopted an adaptive strategy characterized by a thickened leaf epidermis and cuticle to minimize transpirational water loss [25,26]. Collectively, these results showed that desert shrubs do not converge on a single anatomical adaptive strategy in such conditions, but instead partition stress tolerance through coordinated, organ-level allocation trade-offs.

The PC2 axis was predominantly characterized by cortex thickness (CT) and was largely independent of the stem–leaf allocation gradient captured by PC1. As most other traits were distributed toward the negative end of this axis, variation in cortical development appears to represent a distinct and broadly shared dimension of anatomical differentiation rather than a simple extension of the leaf-stem trade-off. This distribution pattern suggests that cortex thickness may contribute to a regulatory and buffering strategy that operates independently of inter-organ allocation [27]. The cortex can function as a reservoir for short-term water and nutrient storage, buffering plants against transient resource limitation [28,29]. This storage capacity, in turn, enhances plant adaptability to environmental fluctuations—particularly those related to water availability and nutrient deficiency. Furthermore, the cortex also contributes to photosynthetic activity, providing additional carbon gain when leaf function is constrained [30]. Overall, this physiological regulation and buffering strategy likely represents a distinct ecological adaptation mode. This mode enables plants to maintain stable physiological functions under highly uncertain environmental conditions.

Taken together, the stem–leaf allocation trade-off represented by PC1 and the cortical buffering dimension represented by PC2 define a multidimensional anatomical strategy space. This framework indicates that desert shrubs do not rely on single-trait adjustments to cope with multiple stresses. Instead, they assemble coordinated suites of anatomical traits across organs, combining allocation trade-offs and buffering mechanisms to enhance persistence under high-altitude arid conditions. Such multidimensional strategy differentiation highlights the importance of anatomical coordination in mediating environmental filtering under extreme environmental conditions.

### 3.2. Responses of Different Functional Groups to Environmental Factors

Environmental analyses further indicated that the two principal axes of anatomical differentiation were governed by different environmental filters (Figure 2). PC1 was associated with temperature, water status, and soil nutrients, suggesting that the primary stem–leaf allocation trade-off emerges from coordinated responses to multiple environmental dimensions. In contrast, PC2 was mainly affected by precipitation-related parameters, implying that cortical development represents a more specific buffering response to variation in water supply. ANOVA was then conducted on the same anatomical trait across different species, revealing no significant difference in the cortex thickness (CT) index (as indicated by the PC2 axis) between the two functional groups (Figure 2). Specifically, Group A showed higher values for stem anatomical traits, while Group B exhibited higher values for leaf anatomical traits. Group-specific correlation analyses further clarified environmental associations (Figure 3). Group A was more strongly associated with aridity index and evapotranspiration, whereas Group B showed stronger relationships with elevation and temperature-related variables, indicating differentiation along a coupled thermal–drought gradient.

For Group A, drought appears to be the primary environmental factor driving the differentiation of its stem anatomical traits. Species in this group were concentrated in lower-elevation sites characterized by higher evapotranspiration and stronger water deficit (Figure 4). Under these conditions, stresses likely impose strong selection on traits that maintain water transport while buffering transient water limitation. Shrubs within this functional group coped with drought stress primarily through two mutually synergistic pathways: enhancing the safety and efficiency of the water transport system and improving water storage. In terms of water transport, high evapotranspiration significantly elevated the water column tension in xylem vessels, thereby increasing the risk of vessel embolism and hydraulic failure [31,32,33]. To mitigate this risk, species in this group exhibited greater central column diameter and xylem thickness, indicating enhanced investment in conductive tissues. Such structural reinforcement may increase hydraulic redundancy and improve resistance to catastrophic conductivity loss, thereby maintaining functional water transport under high tension [34,35]. At the same time, thicker pith tissues may improve internal water buffering. Specifically, pith cells contain specialized parenchymatous water-storing cells [36] that rapidly accumulate water when available and slowly release it during periods of intense transpiration [37]. This storage-release dynamic may reduce short-term fluctuations in tissue water status and reduce the risk of hydraulic disruption during episodic drought.

Notably, this pattern contrasts with responses reported for herbaceous plants, in which stem and pith diameters often decrease under drought [38,39]. This contrast suggests that growth form may strongly influence drought-response strategies. Shrubs appear to adopt a drought-tolerance strategy by reinforcing transport and storage tissues to withstand persistent water limitation, whereas herbs may more often rely on drought avoidance by reducing transpiring surface area and limiting water loss [39,40]. Taken together, these results indicate that Group A adopts an integrated stem-based drought strategy that integrates hydraulic maintenance with internal water buffering, thereby enhancing resilience to chronic water deficit and high evaporative demand.

For Group B, its anatomical traits exhibited a distinct adaptive tendency toward high-altitude environments. Species in this group were concentrated at higher elevations characterized by lower temperatures and intensified radiation exposure (Figure 4). This distribution indicates that altitude-associated stressors, particularly cold and intense radiation rather than drought intensity, represent the dominant selective forces shaping their trait configuration. In this context, thicker cuticles and epidermal tissues likely provide dual protective benefits. First, increased ultraviolet radiation at high altitude promotes thickening of the cuticle and epidermal tissues, enhancing photoprotection and reducing radiation-induced cellular damage [41,42]. Second, low-temperature stress further enhanced the development of the cuticle and epidermis [43,44], helping to buffer metabolically active internal tissues against thermal stress and to maintain physiological function under cold conditions [45]. Accordingly, Group B appears to adopt a leaf-centered conservative strategy that prioritizes tissue protection and transpiration control under combined cold and radiation stress. By allocating resources toward epidermal and cuticular development, these species enhance physiological stability in high-altitude environments where thermal limitation and photoinhibition constrain growth.

This interpretation is broadly consistent with studies from other high-altitude ecosystems, where leaf thickness often increases with elevation and is thought to enhance tolerance to cold and radiation stress by protecting photosynthetically active tissues such as the mesophyll [46,47]. However, opposite patterns have also been reported. For example, some studies have found that leaf thickness declines with elevation [48]. Such discrepancies likely arise from the differences in the environmental context of the elevational gradient. In relatively low-elevation and wetter systems, where precipitation increases with altitude and plants are not simultaneously exposed to severe drought and intense radiation, selection for structurally reinforced leaves may be weaker [48]. Under those conditions, species may instead reduce biomass investment in leaf construction and rely more on alternative mechanisms, such as osmotic adjustment, to cope with low temperature or radiation stress [48].

Our results show that anatomical divergence between high- and low-altitude arid areas reflects a shift in dominant environmental filters rather than differences in aridity alone. In lower-elevation deserts, species exhibited greater investment in stem conductive tissues, including enlarged central cylinders and thicker xylem, consistent with a hydraulic reinforcement strategy adapted to high temperature and elevated evaporative demand. In contrast, shrubs from higher elevations allocated proportionally more biomass to leaf protective and assimilatory tissues, including thicker cuticles and epidermal layers, suggesting enhanced insulation and radiation buffering under cold and high-radiation conditions. Partial correlation analyses further showed that this differentiation was primarily structured by thermal and evaporative gradients rather than soil nutrient availability, with elevation acting indirectly through its association with temperature and atmospheric water demand (Table 1). Together, these findings demonstrate that anatomical trait coordination in desert shrubs arises from multidimensional environmental filtering, where hydraulic safety dominates at low elevations, whereas tissue protection and thermal buffering become critical at high elevations. This shift underscores that arid-zone adaptation is not uniform but reorganized by the interaction of water limitation with temperature and radiation stress.

To further resolve how anatomical traits mediate desert shrub adaptation to multiple stresses, future studies should focus primarily on the following two aspects. First, the current results are primarily focused on the anatomical structure level and lack associations with key plant physiological functions (e.g., photosynthetic rate and water-use efficiency). Because anatomical organization ultimately constrains processes such as carbon assimilation, water-use efficiency, and hydraulic function, integrating multi-scale observations of tissue structure, gas exchange, and plant water relations will be essential for establishing the functional consequences of anatomical differentiation. Such integration would substantially improve predictions of vegetation dynamics in arid ecosystems. Second, this study primarily relies on field sampling, which is insufficient to adequately capture the long-term adaptive responses of plant adaptive strategies to climate change. Integrating observational gradients with manipulative experiments, such as warming and precipitation treatments, would allow direct assessment of strategy resilience, potential trait reorganization, and community-level shifts under altered thermal and hydrological regimes. Such efforts are necessary to forecast how arid shrub communities will restructure as drylands continue to expand.

In summary, desert shrubs exhibit distinct differentiation in anatomical traits and resource allocation between stem and leaf tissues. Species in Group A allocate more resources to stem conductive and storage tissues, enhancing hydraulic safety and water transport capacity under warmer and more evaporative conditions. In contrast, species in Group B predominantly invest in protective leaf structures, including thicker epidermal and cuticular tissues, consistent with a defense strategy against cold stress and intense radiation at high elevations. These contrasting strategies reflect coordinated organ-level specialization shaped by distinct environmental filters. Rather than representing uniform drought adaptation, desert shrubs partition adaptive investment across tissues in response to multidimensional stress gradients. This divergence highlights how environmental heterogeneity reorganizes trait coordination and drives functional differentiation in arid ecosystems.

## 4. Materials and Methods

### 4.1. Study Area

This study was conducted in the Qaidam Basin, a vast endorheic depression on the northeastern Tibetan Plateau. Characterized by an arid to semi-arid climate, the basin extends approximately 1000 km east–west with a width of 200–400 km. The specific study area lies between 90°13′–99°01′ E and 35°55′–38°40′ N, with an altitude ranging from 2681 m to 3750 m. The region has a plateau continental climate [49], with an annual mean temperature ranging from −1.61 °C to 5.46 °C and an annual precipitation of 34 mm to 268 mm. Soil types are dominated by brown calcic soil, solonchak, and aeolian sandy soil [50]. Desert steppe and typical desert are the dominant vegetation types, with predominant species including *Oreosalsola abrotanoides*, *Nitraria tangutorum*, *Krascheninnikovia ceratoides*, and *Reaumuria songarica*.

### 4.2. Large-Scale Field Sampling

Samples were collected from desert ecosystems in the Qaidam Basin during July–August of 2019–2023, with sampling restricted to sites experiencing minimal anthropogenic disturbance. Sampling sites were systematically selected based on the 1:1,000,000 Vegetation Map of China [51] to ensure the representation of key vegetation types (desert steppe and typical desert). To minimize the influence of spatial autocorrelation, a minimum separation distance of 10 km was maintained between adjacent sites. Furthermore, the intentional avoidance of vegetation patches dominated by a single species in proximate areas was designed to adequately reflect the diversity of plant community types.

**Figure 5:**
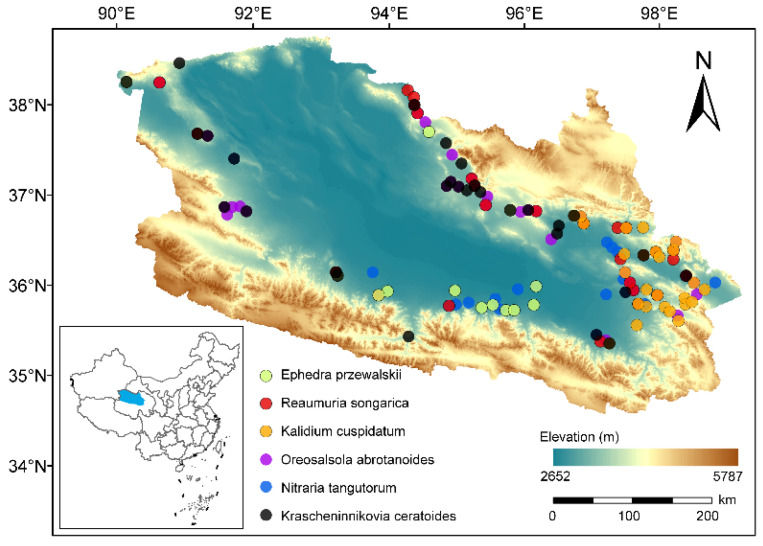
Distribution of sampling sites for six dominant desert species in the Qaidam Basin. Point colors correspond one-to-one to the six species listed in the legend. The background shows elevation (m), and the inset indicates the location of the study area within China.

In line with the principles outlined above, this study surveyed 100 representative sample sites (Figure 5). At each site, plots measuring either 5 m × 5 m or 10 m × 10 m were established, and the latitude, longitude, altitude, along with other relevant information, was accurately recorded using GPS technology. Within each plot, three healthy specimens of the dominant or constructive species were chosen for sampling (replicates of plants sampled at each site = 3). From the upper and middle canopy of each specimen, 3–4 mature, healthy, and undamaged branches were collected to ensure that the sampled branches were exposed to relatively consistent environmental conditions, such as light and moisture. All samples were collected during clear mornings to minimize physiological fluctuations throughout the day. After collection, the specimens were immediately placed in sealed sampling bottles containing FAA fixative solution (100 mL FAA = 90 mL 70% ethanol + 5 mL 37% formaldehyde + 5 mL 99.5% glacial acetic acid), stored at low temperatures, and transported to the laboratory at the earliest opportunity for subsequent anatomical trait analysis.

### 4.3. Study Species

Six dominant desert plant species from the Qaidam Basin (four families) were selected (Table 2). All species exhibit xerophytic adaptations including reduced leaf surface area. Four species have succulent leaves for water storage (*R. songarica*, *N. tangutorum*, *K. cuspidatum*, and *O. abrotanoides*), while two species possess spinescent structures (*N. tangutorum*, *O. abrotanoides*). Divergent strategies include: extreme leaf reduction with stem photosynthesis in *E. przewalskii*; dense stellate trichomes in *K. ceratoides*; and salt-secreting glands in *R. songarica*. Life forms comprise two shrubs, two dwarf shrubs, and two subshrubs.

### 4.4. Measurement of Anatomical Traits

**Figure 6:**
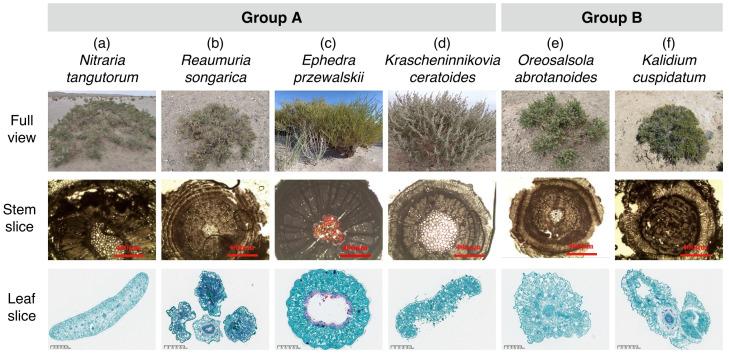
Morphological and anatomical characteristics of six dominant desert shrubs in the Qaidam Basin. The species are categorized into two functional groups (Group A and Group B) based on their adaptive strategies. Group A includes (**a**) *Nitraria tangutorum*, (**b**) *Reaumuria songarica*, (**c**) *Ephedra przewalskii*, and (**d**) *Krascheninnikovia ceratoides*. Group B includes (**e**) *Oreosalsola abrotanoides* and (**f**) *Kalidium cuspidatum*. For each species, the top, middle, and bottom panels show the whole-plant view, stem transverse section, and leaf transverse section, respectively. Red scale bars in stem sections represent 400 μm; black scale bars in leaf sections represent 200 μm.

In this study, the anatomical traits of plant leaves and stems were examined primarily using paraffin sectioning and microscopic observation techniques (Figure 6). Eight anatomical traits were quantified, comprising four leaf and four stem traits. The leaf anatomical traits included cuticle thickness (Ct), upper epidermal thickness (ET_up_), lower epidermal thickness (ET_low_), and mesophyll thickness (or assimilation branch parenchyma thickness) (MT). The stem anatomical traits included cortex thickness (CT), central cylinder diameter (CCd), xylem thickness (XT), and pith thickness (PT).

Following sample collection, all specimens underwent a standard processing sequence: dehydration through a graded ethanol series, clearing, paraffin infiltration and embedding, sectioning, and staining with safranin and fast-green. The prepared slides were observed and photographed using an optical microscope (N-117 M, Ningbo Yongxin Optical Co., Ltd., Ningbo, China). All trait measurements were performed on the captured micrographs using the ImageJ software (version 15.4n 17; National Institutes of Health (NIH), Bethesda, MD, USA). To ensure data accuracy, each trait was measured in three randomly selected fields of view per sample slice, and the mean value was calculated as the final data.

### 4.5. Environmental Factor Data

The environmental factors selected in this study were classified into three categories: temperature characteristics, water and aridity conditions, and soil nutrient properties. Temperature-related variables included five indicators (i.e., MAT, TCQ, and TWQ). Water and aridity conditions were represented by seven variables, including water status (i.e., MAP, PDQ), aridity index, and evapotranspiration. Soil nutrient properties comprised five variables such as total nitrogen (TN) and total phosphorus (TP). Climatic, water and aridity data were retrieved from the WorldClim database (https://worldclim.org, accessed on 20 August 2025) at a spatial resolution of 2.5 arcminutes, while soil nutrient data were acquired from the SoilGrids database (https://soilgrids.org, accessed on 20 August 2025) with a spatial resolution of 250 m [52].

### 4.6. Statistical Analysis

First, to examine differences in stem and leaf anatomical traits among the six shrub species and between the two functional groups, we performed ANOVA using species identity and functional group as fixed factors. Second, to assess relationships between anatomical differentiation and environmental gradients, we calculated Pearson’s correlation coefficients between trait PC1 and PC2 scores and individual environmental variables, including elevation, temperature-related variables, water status-related variables, evapotranspiration (ET), aridity index, and soil nutrient indicators. As environmental variables within each category were potentially collinear, we conducted separate PCA for temperature-, water status-, and soil-related variables. Temperature variables included MAT, TWEQ, TDQ, TWQ, and TCQ. Moisture-related variables included MAP, growing-season SWC, PWEQ, and PDQ. Soil-related variables included STN, STP, CN, and NP. The PC from each environmental PCA was used as an integrated descriptor of temperature, moisture, and soil conditions. Linear regressions were then conducted between trait PC1/PC2 and the corresponding environmental PC1 scores.

Third, to determine whether the relationship between trait PC1 and elevation was independent of other environmental factors, we performed partial correlation analyses. Pearson’s correlation between trait PC1 and elevation was first calculated without covariates, followed by models sequentially controlling for temperature-related, moisture-related, aridity, evapotranspiration, and soil nutrient variables, both individually and in combination. Finally, to compare environmental conditions among the six species and the two functional groups, we focused on elevation, Growing-season ET, and MAT. We further examined the relationships between elevation and MAT, and between MAT and ET, using linear regression.

All statistical analyses were conducted in R (v.4.4.1) [53]. PCA was performed using the vegan package (version 2.6-8) [54], Pearson correlations using the psych package (version 2.6.3) [55], partial correlations using the ppcor package (version 1.1) [56], and figures were generated with ggplot2 package (version 4.0.2) [57].

## 5. Conclusions

By integrating large-scale field surveys with detailed anatomical trait measurements, we investigated stem and leaf variation across six dominant desert shrub species in the Qaidam Basin and identified the environmental drivers underlying these patterns. Our results highlight two key adaptive strategies. First, survival in high-altitude arid ecosystems depends on organ-specific specialization rather than whole-plant uniformity. We observed a trade-off between leaf protective traits and stem hydraulic-storage capacities, suggesting that different plant organs specialize to mitigate specific stressors. Second, environmental filtering modulates resource allocation. At higher elevations characterized by intense radiation and low temperatures, species prioritize reinforced leaf protection. Conversely, under warmer and high-evaporative conditions, investment shifts towards stem hydraulic safety and water storage to mitigate drought stress. Collectively, these findings demonstrate that desert shrubs employ multifaceted adaptive mechanisms to cope with environmental stressors, providing a theoretical basis for predicting vegetation responses and informing ecological restoration in high-altitude arid ecosystems.

## Figures and Tables

**Table 1:** Partial correlation analysis between Trait PC1 and altitude under different environmental controls.

Item	Controlling Factor	*p* Value
Trait PC 1 vs. altitude	None	<0.001
	MAP	<0.001
	MAP + PWEQ + PDQ + SWC	0.017
	MAT	0.049
	MAT + TWEQ + TDQ + TCQ	0.067
	ET	0.424
	AI	<0.001
	STN + STP + NP + CN	0.004

Abbreviations—MAP, mean annual precipitation; PWEQ, precipitation of the wettest quarter; PDQ, precipitation of the driest quarter; SWC, growing season soil water content; MAT, mean annual air temperature; TWEQ, mean temperature of the wettest quarter; TDQ, mean temperature of the driest quarter; TCQ, mean temperature of the coldest quarter; ET, growing season evapotranspiration; AI growing season aridity index; STN, total nitrogen; STP, soil total phosphorus; NP, nitrogen-to-phosphorus ratio; CN, carbon-to-nitrogen ratio.

**Table 2:** Taxonomic classification, life forms, and stem and leaf characteristics of the six studied shrub species.

Group	Species	Family	Genus	Life Form	Stem	Leaf
A	*Krascheninnikovia ceratoides*	Amaranthaceae	*Krascheninnikovia*	Subshrub	Densely covered with yellowish-brown stellate trichomes	linear or lanceolate
	*Ephedra przewalskii*	Ephedraceae	*Ephedra*	Shrub	Woody stem with green photosynthetic branches	Reduced to membranous sheaths
	*Reaumuria songarica*	Tamaricaceae	*Reaumuria*	Dwarf shrub	Bark irregular, peeling in thin flakes	Succulent cylindrical, with salt-secreting glands
	*Nitraria tangutorum*	Nitrariaceae	*Nitraria*	Shrub	Multi-branched, branch tips spine-like	Succulent, oblanceolate
B	*Kalidium cuspidatum*	Amaranthaceae	*Kalidium*	Dwarf shrub	Young branches yellowish-green	Ovate, succulent
	*Oreosalsola abrotanoides*	Amaranthaceae	*Oreosalsola*	Subshrub	Young branches with fine longitudinal ridges	Semi-cylindrical, apex with hard spines

## Data Availability

The data that support the findings of this study have been deposited in the Figshare public repository, and are accessible via DOI: 10.6084/m9.figshare.31747321.
